# Do you Hear what I Hear? A Qualitative Study Examining Psychological Associations Underlying Evaluations of Everyday Sounds in Patients with Chronic Tinnitus

**DOI:** 10.3390/jpm13040690

**Published:** 2023-04-20

**Authors:** Christina Baniotopoulou, Benjamin Boecking, Birgit Mazurek

**Affiliations:** Tinnitus Center, Charité Universitätsmedizin Berlin, 10117 Berlin, Germany; christina.baniotopoulou@charite.de (C.B.); benjamin.boecking@charite.de (B.B.)

**Keywords:** associations, neutral sounds, qualitative design, tinnitus

## Abstract

Tinnitus is a multifactorial phenomenon and psychological, audiological, or medical factors can facilitate its onset or maintenance. A growing body of research investigates individuals’ perceptions, associations, and experiences of living with tinnitus. This body of research examines tinnitus as a condition rather than a symptom. We examine a sample of chronic tinnitus patients in terms of associations that are induced by neutral sounds. In particular, we investigate how patients with chronic tinnitus ascribe meaning to those neutral sounds. The present study uses Mayring’s content analysis to explore the content of psychological associations underlying valence ratings of everyday neutral sounds. Nine tinnitus patients completed a hearing exercise, during which they listened to seven neutral sounds, following which we examined their sound-induced associations using semi-structured interviews. Three groups of factors influenced patients’ associations and valence ratings of neutral sounds: affect, episodic memory, and ‘other’. The former two factors further comprised two subcategories. In line with previous psychoaudiological research designs, our findings suggest that neutral, everyday auditory stimuli evoke strong affective reactions—possibly through serving as retrieval cues for episodic memories. Based on these findings, we discuss our results in the context of previous psychoaudiological findings and propose further research concerning psychological associations that may specifically underlie the tinnitus sound.

## 1. Introduction

Tinnitus denotes the experience of noise or a sound without corresponding acoustic sources [1]. It is a complex, multifactorial phenomenon, and psychological, audiological, or medical factors can facilitate its onset or maintenance [2]. Psychological mediators of tinnitus maintenance and tinnitus-related distress include depression [3], anxiety [4], as well as perceived stress [5].

Further, dispositional personality traits can mediate tinnitus-related distress following symptom onset. For example, some patients with chronic tinnitus show higher levels of neuroticism [6] and lower levels of extraversion [7]. However, possibly owing to the patient population’s heterogeneity [8], not all studies suggest personality conspicuities [9].

Whilst relationships between different psychological factors and the chronic tinnitus experience have been intensively examined in the last years, the exact nature of involved interactions remains unclear. A growing body of qualitative designs set out to investigate individuals’ perceptions of the tinnitus sound and its relation to tinnitus-related distress from a patient’s point of view.

Watts et al. [10] analysed patients’ free-text responses to investigate why tinnitus was perceived as a problem. Results revealed eight domains of tinnitus-related distress, the most important of which comprised reduced “quality of life”, “fear”, “constant awareness”, “annoyance”, and “inability to concentrate”.

Andersson and Edvinsson [11] explored the tinnitus experience in subjects involved in psychological treatment for chronic tinnitus. Using semi-structured interviews, the material was analysed following principles inspired by the grounded theory. Their results revealed emotional ambivalence towards tinnitus as a driving force of tinnitus-related distress and three main themes emerged: (1) consequences of tinnitus (mainly negative consequences of living with tinnitus), (2) treatment experience (a summary of treatment experiences in the department of audiology/other providers of care as well as patients’ ambivalence towards knowledge), and (3) tinnitus identity (the ways tinnitus affects one’s private and public identity).

Colagrosso et al. [12] used qualitative research to assess factors that modulated patients’ individual tinnitus experiences. Using data derived from semi-structured interviews and journal entries, three main themes emerged: (1) participants’ appraisal of their tinnitus, (2) the consequences of tinnitus, as well as (3) environmental and individual factors of attentional focus, stress level, physical state, fatigue, and auditory effort. They identified a dissociation between the tinnitus sensation (i.e., objective loudness, pitch, timbre) and patients’ associated reactions (i.e., perceived loudness, distress) and suggested that this dissociation could potentially have important clinical implications regarding individuals’ ability to cope with the percept.

Pryce and Chilvers [13] examined notions and interpretations of tinnitus patients regarding the tinnitus symptom, as well as their experience of living with it. Here, qualitative analyses revealed that tinnitus experiences—including the symptom’s acceptance—were influenced by patients’ thinking patterns and “need for sense making”, i.e., rationalising tinnitus and gaining an understanding of its impact on one’s life. The authors postulated that sense making is an important step towards symptom acceptance.

A recently published paper [14] discussed the burden of illness theory as a framework to understand the allocation of health resources. This theory describes the different ways the health system transfers accountability to patients in order to manage long-term conditions. Using qualitative thematic analysis, the authors examined patients’ accounts of their experiences with living with tinnitus as well as treatments undertaken. Their results showed that the burden of tinnitus could be understood as (1) an effort to cope with the interference of tinnitus in everyday life, as well as (2) behavioural adjustments and modifications that minimise its interference. They additionally identified (3) changes in self-perception after the onset of tinnitus, as well as (4) efforts to integrate those changes in everyday life as relevant characteristics of the burden of tinnitus. The authors proposed that understanding the psychological burden for those living with the condition will further aid the formation of patient-centred interventions.

Dauman and Dauman [15] interviewed 21 tinnitus patients regarding tinnitus intrusiveness. Using a qualitative analysis method, they identified patterns that influenced tinnitus-induced frustration: (1) searching for perspective on tinnitus, (2) controlling one’s perception despite the tinnitus intrusiveness, and (3) reducing conflicts that arise from social interactions were key patterns which they identified. They incorporated these findings in an empowerment model, which emphasized addressing feelings of frustration and encouraging participation in meaningful activities.

Another qualitative study [16] set out to investigate attachment style, perceived family support, and tinnitus-related problems. Results demonstrated that avoidant attachment was linked to tinnitus-related problems and that this attachment style was further associated with perceived punitive family responses. The authors argued that these preliminary results could shed further light on the interactions between coping style, perceived support, and tinnitus-related distress.

Erlandsson et al. [17] used a qualitative design to explore interactions between tinnitus and unique life histories. They interviewed five tinnitus patients using unstructured interviews with free conversation, focussing on the patients’ narrative styles. Their results showed that “regressive” narratives (i.e., including themes suggesting deprivation of basic psychological needs such as autonomy, competence and relatedness) demonstrated an alienation from valued life goals, while “progressive or stable” narratives indicated vitality and orientation in life. They further argued that narrative styles carry important information about patients’ coping styles as well as their resources and should therefore be clinically addressed.

Whilst all above-mentioned studies highlight the importance of investigating individuals’ perceptions and the content of tinnitus-related associations, the studies examined patients’ experiences with tinnitus and patients’ associations induced by tinnitus, regarding tinnitus as a condition, not explicitly as a sound stimulus. This constitutes a gap in our understanding of the interactions between tinnitus as an acoustic phenomenon and linked psychological associations.

The aim of the present pilot study was to explore (1) associations linked to everyday sounds in a group of patients with chronic tinnitus and (2) possible influencing factors of valence ratings. Ten participants (nine of whom provided analysable data) completed a hearing exercise wherein seven neutral auditory stimuli (e.g., a match being lit; applause) were rated as “positive”, “neutral”, or “negative”. In a semi-structured interview, patients’ individual associations with these sounds were further explored. All conversations were transcribed and analysed using Mayring’s qualitative content analysis method.

## 2. Materials and Methods

### 2.1. Participants

Ten participants (between 26 and 78 years) were sampled from the patient population of the Tinnitus Center, Charité Universitaetsmedizin Berlin (See Table 1 for an overview of demographic characteristics). Inclusion criteria were [1] age over 18 years old and meeting criteria for [2] a diagnosis of chronic tinnitus (with the tinnitus symptom persisting for ≥ 3 months). Exclusion criteria included [1] a diagnosis of hyperacusis or [2] moderate–severe hearing impairment as defined by the World Health Organization (WHO) (audiometric ISO value > 40 dB) [18]. The study was reviewed and approved by the Ethics Committee of the Charité, Universitätsmedizin Berlin (EA4/217/20). Participants provided their written informed consent to participate in this study and all principles outlined in the Declaration of Helsinki were met.

### 2.2. Procedure

#### Hearing Exercise

Participants individually completed the hearing exercise in a room with little visual distraction. Before the hearing exercise, they were welcomed and instructed on how to perform the test. During the hearing exercise, each participant listened to seven neutral auditory stimuli once (standard volume: 20 dB). Stimuli included taking off of an airplane [1], starting a car engine [2], a creaking door [3], a ticking clock [4], a match being lit and blown out [5], birds chirping [6], and applause [7]. Using a plain worksheet, participants were asked to note down any associations evoked as a response to each sound, as well as to rate the stimulus as pleasant [1], neutral [2], or unpleasant [3].

### 2.3. Interviews

Following the hearing exercise, a semi-structured interview further explored participants’ associations and identified potential influencing factors of participants’ valence ratings (See Table 2 for an overview of the interview questions). The average duration of the interviews was 60 min. All interviews were audio-recorded and subsequently transcribed.

The semi-structured interview was conceptualised according to principles derived from episodic interviewing, which focuses on individuals’ perceptions of a given subject. Episodic interviewing invites participants to recall and describe situations in their lives that are relevant to the given subject (so-called “episodes”) [19]. Questions in the semi-structured interviews covered four main thematic areas: (1) identification of spatiotemporal characteristics of the association (i.e., where and when characteristics), (2) emotions evoked by the auditory stimulus, (3) thoughts evoked by the auditory stimulus, and (4) factors that influenced the valence rating of the auditory stimulus.

In addition, a few follow-up questions were prepared in order to clarify associations in terms of place and time, as applicable (e.g., When you think of this association, where were you?/When you think of this association, could you classify it chronologically?).

### 2.4. Data Analysis

One interview was not transcribed due to poor audio quality. The remaining nine interviews were transcribed verbatim and analysed using Mayring’s systematic method of qualitative content analysis. Qualitative content analysis defines itself “as an approach of empirical, methodological controlled analysis of texts within their context of communication, following content analytical rules and step by step models” [20]. Central to Mayring’s content analysis methodology is the development of a category system that stems directly from the corpus data [21].

Based on the respective research design, this methodology offers the option of inductive or deductive category formation. Because the present study was exploratory, an inductive category formation was chosen. Data analysis comprised the following steps:

[1] The formulation of the research question “Which individual factors do participants name when describing sound-induced associations, whilst valence-rating a neutral auditory stimulus?”
[2] The determination of the category definition, i.e., subjective factors relevant in the process of evaluating a neutral auditory stimulus
[3] The definition of the level of abstraction, which was defined as concrete individual factors, with no general statements. The content analytical units were:
  [1] Recording unit: all 9 interviews;
  [2] Context unit: the whole transcribed interview;
  [3] Coding unit: clear semantic elements in the text.

**Figure 1:**
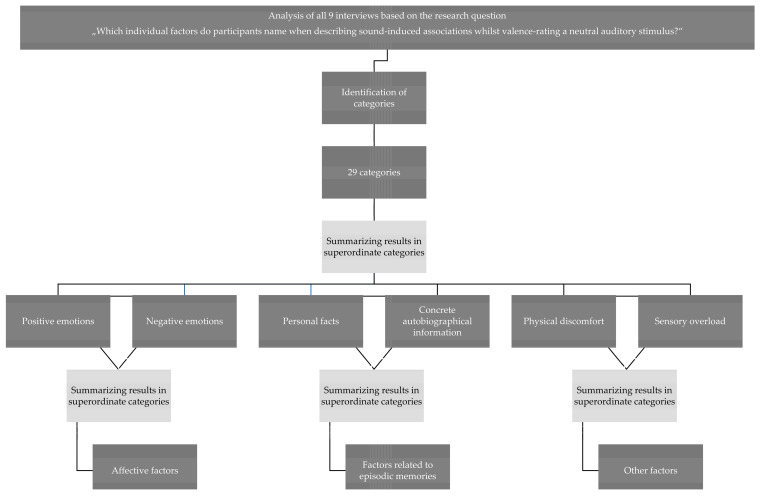
Data analysis process based on Mayring’s systematic method of qualitative content analysis.

During the first phase of the analysis, all 9 interviews were analysed line by line. During this phase, relevant information was identified, and categories were created. Every time new relevant material was detected, this was either subsumed under an existing category or a new category was created. During the second phase of analysis, the identified categories were thematically summarised. During the third phase of analysis, factors were summarised into superordinate thematic categories (see Figure 1 for an overview of the analysis process).

## 3. Results

Following the first phase of data analysis, 29 categories emerged, which described a variety of factors that coded psychological associations that were induced by the given neutral sounds (See Table 3 for an overview). These categories were subsequently summarised into major thematic categories labelled [1] affective factors, [2] factors related to episodic memories, and [3] other factors.

### 3.1. Affective Influences

Affective factors appeared to shape participants’ associations to neutral sounds when rating a stimulus’s valence. Among the identified affective influences, feelings of (1) fear, (2) threat, (3) pressure, and (4) pleasure occurred most frequently among the interviews. See Table 4 for examples of the identified affective factors and corresponding associations:

### 3.2. Episodic Memories

Examples of episodic memory-related influences (see Table 5 for a list of examples) in the listening to and rating of the neutral sounds were:

### 3.3. Other Factors

Other factors inherent in sound-induced associations related to (1) physical discomfort and (2) sensory overload (see Table 6 for a list of examples):

## 4. Discussion

The present study set out to qualitatively explore sound-induced psychological associations in a sample of patients with chronic tinnitus. Results indicated three main categories that influenced sound-induced associations: “affective factors”, “factors related to episodic memories”, and “other factors” (i.e., physical discomfort and sensory overload). Our findings suggest that psychological associations induced by neutral auditory stimuli are intricately linked with emotional states that further influence the valence rating and “experience” of those stimuli. Our findings are in line with previous research which demonstrates that auditory stimuli can evoke emotional reactions [22], which are further central to the perception and categorisation of everyday sounds [23]. In keeping with a previous study that suggested episodic memories as links between musical experiences and emotional reactions [24], our analysis further showed that neutral sounds were linked to episodic memories, that is, personal facts and concrete autobiographic information, which, in turn, influenced the sounds’ valence rating. A review investigating how music was associated with memory suggested that music serves as a retrieval cue for autobiographic information and induces memories of associated events [25]. In line with this suggestion, we propose that everyday sounds likewise serve as retrieval cues and that the autobiographic information that arises influences the subject’s interpretation and valence rating of a neutral sound. A growing body of research has set out to understand the subjective interpretation of sound and the factors that mediate the individual affective response to sounds. Asutay et al. [26] proposed that individuals react to “auditory events” and argued that apart from the objective characteristics of the sound, both the sound’s context and the listener are integral parameters in any emotional reactions to sounds. In particular, the meaning a listener ascribes to a sound stems from his/her associations and previous experiences. This line of argumentation agrees with our findings, which showed a variety of combinations of different psychological associations and corresponding ratings of the same neutral auditory stimuli. We therefore assume that the rating of a neutral auditory stimulus as pleasant/neutral/unpleasant is mediated by participants’ subjective interpretations of the sound. The above findings could shed light on the different ways tinnitus patients psychologically react to the tinnitus sound.

Previous research has examined the interactions between tinnitus loudness and tinnitus-related distress [27], as well as mediating factors between perceived loudness and tinnitus-related distress [28]; future studies should investigate the psychological associations induced by tinnitus as an acoustic stimulus, and the way these associations mediate tinnitus perception and distress. Neuropsychological studies of tinnitus have shown that both acoustic and non-acoustic systems in the brain are involved. Using imaging techniques, increased activity has been measured in both the primary and secondary auditory cortex, as well as in the limbic system and frontal brain [29]. The activation of brain areas responsible for auditory and cognitive/emotional processing suggests that tinnitus could be understood both as an auditory stimulus and as a psychological retrieval cue. Future studies should explore the subjective interpretation of the tinnitus sound, as well as identify subjective factors that mediate tinnitus perception and its rating.

The present design has several limitations: first, whilst the small sample size might prevent the generalisation of the results, the present study understands itself as an initial exploration of participants’ associations to neutral sounds in a population of patients with chronic tinnitus. Since this paper serves as a proof of concept (PoC) study for upcoming research on possible interactions between tinnitus as an audiological stimulus and underlying affective responses, our main aim was to initially identify psychological factors that mediate the general experience and valence ratings of auditory stimuli in a tinnitus population. Based on the literature concerning the sample size in qualitative designs, the nature of the research question, and the aim of our study, we propose that ten participants offer enough information power for this pilot study design [30]. Future research will include larger sample sizes.

Moreover, although Mayring’s qualitative content analysis is a methodologically controlled and structured method, subjective interpretation is one of its central components. Therefore, the above-described results necessarily reflect the rater’s subjectivity. Last, the identified sound-induced associations may have been partly biased by the phrasing of the interview questions.

Despite these drawbacks, the present study is the first to explore psychological associations induced by everyday sounds in a sample of patients with chronic tinnitus. Psychodynamic-focused research describes tinnitus as a cry of the soul [31]. Tinnitus “takes over” the role of the messenger; one could hypothesise that tinnitus has a dual character: a “real” somatic one, as well as a symbolic one. The symbolic meaning of the tinnitus is interconnected with the biography of the individual, and thus cannot be generalised. Therefore, an exploration of the psychological associations induced by the tinnitus sound could potentially enhance our understanding of the ways auditory and psychological parameters interact.

The aim of the study was not to identify similarities and/or differences in the sounds’ effects between a tinnitus population and patients without tinnitus but rather to explore the process of hearing everyday sounds and those sounds’ effects explicitly in a tinnitus population. The present design is not in its nature a case–control, it is much more an initial attempt to explore the process of how everyday sounds are heard and perceived in a tinnitus population. It is not assumed that this process necessarily differs in patients with chronic tinnitus as compared to healthy controls. Rather, the study sets the stage for further studies into how tinnitus sound is linked to autobiographical memories, therefore, opening up potentially important psychological treatment avenues to move the tinnitus sound towards being experienced in a more benign manner. Using a qualitative design and choosing an inductive category development process, we aimed to identify psychological factors that underlie the valence rating of everyday sounds. Furthermore, this is a proof of concept (PoC) study aiming to initially assess aspects of the complex interactions between auditory and affective parameters underlying the valence rating of everyday sounds. Based on our preliminary results, we will prospectively explore how psychoauditive interactions affect the way that tinnitus patients ascribe meaning to the tinnitus sound.

Overall, whilst the qualitative design of the present study bares some limitations, we emphasise the importance of qualitative research to expand our understanding of the tinnitus experience and individuals’ tinnitus interpretations. As proposed by a case study conducted by Dauman and Erlandsson [32], articulating an individual’s tinnitus experience and forming a coherent narrative may be central to facilitating psychosomatic interactions and positively affect symptom-related distress. The authors additionally stress the clinical importance of understanding tinnitus-related distress from a patient’s point of view and incorporating the patient’s narrative into psychological intervention frameworks.

Furthermore, there is an ongoing shift within evidence-based therapies away from diagnosis-driven interventions and symptom-specific protocols and towards personalised interventions that aim to meet the specific needs of the individual [33]. To further personalise tinnitus interventions, it is first important to explore and analyse the experiences of tinnitus sufferers and to filter out common denominators. Qualitative research designs enable an in-depth exploration of the subjective experience of living with tinnitus.

To conclude, exploring the subjective interpretation of the tinnitus sound could help us better understand the complex ways patients ascribe meaning to their tinnitus, thus expanding our understanding of the individual’s tinnitus-related distress.

## Figures and Tables

**Table 1:** Demographic characteristics of the participants. Note that due to a recording error, Interview 1 was not included in the final analyses.

Participant	Age	Sex
Interview 1	59	Female
Interview 2	54	Female
Interview 3	58	Female
Interview 4	52	Female
Interview 5	59	Female
Interview 6	39	Male
Interview 7	57	Male
Interview 8	78	Female
Interview 10	62	Male

**Table 2:** List of the interview questions.

Could you identify the auditory stimulus you heard?
What was the association that was evoked when listening to the auditory stimulus?
Can you classify your association in terms of location?
Can you temporally classify your association?
Was it a one-time or repeated association/experience?
Could you describe your emotions when you recall this association/experience?
Could you describe your thoughts when you recall this association/experience?
How did the sound itself affect the rating of the auditory stimulus?
How did the recalled association/experience affect the rating of the auditory stimulus?

**Table 3:** Categories pertaining to participants’ psychological associations of everyday sounds and total coding frequency.

	Categories	Total Coding Frequency
C1	Discomfort	3
C2	Repeated negative experience	9
C3	Fear	15
C4	Abstract autobiographical information	3
C5	Threat	12
C6	Annoyance	5
C7	Feelings of pressure	8
C8	Pleasure	7
C9	Physical discomfort	1
C10	Repeated neutral experience	2
C11	Anger	2
C12	Excitement	2
C13	Personal Fact	17
C14	Relaxation	3
C15	Relief	2
C16	Panic	1
C17	Specific past event	2
C18	Sense of loss of control	2
C19	Sensory overload	2
C20	Sense of achievement	1
C21	Fright	3
C22	Concrete autobiographical information	8
C23	Restlessness	2
C24	Sense of freedom	2
C25	Sense of responsibility	3
C26	Sense of guilt	2
C27	Critique	2
C28	Sense of achievement	2
C29	Repeated positive experience	1

Note. C, category.

**Table 4:** Identified affective factors and corresponding associations.

**Fear**	
**Sound**	
Taking off of an airplane	“*…it is always this fear that if this goes wrong, the plane will crush in here and then it’s all over*” Interview 1
starting Car engine	“*No, I can’t stand that sound, because it is very scary for me again*” Interview 3
	“*I associate driving off in a sudden way with a fear inside me*” Interview 4
**Threat**	
**Sound**	
Creaking door	“*I have to get myself to safety; (otherwise) something could happen*” Interview 1
Taking off of an airplane	“When the airplane is taking off, I am holding on tight, it is threatening to me” Interview 5
	“It is unpleasant, because it is a threat” Interview 6
**Feelings of pressure**	
**Sound**	
Ticking clock	“The ticking clock triggers me, time pressure is always there” Interview 1
	“It is not pleasant, because … the time … being under pressure, it is not pleasant” Interview 6
Starting car engine	“*I associate it with stress, with car driving, with traffic jam, I totally stress myself, because I don’t like it, to arrive too late, and so I put myself under pressure*” Interview 9
**Pleasure**	
**Sound**	
Match being lit and blown out	“Because I simply find it pleasant, nice, it is warming. Somehow for me this sound is insanely pleasant” Interview 1
	“This is pleasant for me, something is being torn open, something that brings me joy” Interview 5
Chirping birds	“*During the week I woke up with an alarm clock, during the weekend it was the chirping birds waking me up, it was extremely pleasant*” Interview 7

**Table 5:** Identified episodic memory-related factors and corresponding associations.

**Personal Facts**	
**Sound**	
Taking off of an airplane	“*This sound is neutral for me, because I live close to an airport*” Interview 8
Chirping birds	“*I associate this with positive feelings, with springtime, spring is my favorite time, also because it is my birthday in spring, I love spring in the city*” Interview 9
**Concrete autobiographic information**	
**Sound**	
Ticking clock	“*I know that from my mother’s house, she had a lot of ticking clocks, and when we spent a weekend at her place, we removed five or six clocks (from the room), we were getting rid of the clocks, because no one can sleep with a ticking clock*” Interview 4
Match being lit and blown out	“*Every time my mother was lighting a candle, it was nice—it was something special*” Interview 8

**Table 6:** Identified other factors and corresponding associations.

**Physical Discomfort**	
**Sound**	
Taking off of an airplane	“*Flying is not so great for me, because my body doesn’t find it so great*” Interview 2
**Sensory overload**	
**Sound**	
Applause/Heavy Rain	“*The louder the rain, the more unpleasant it becomes, because the volume... I don’t know exactly; it is again just too much*” Interview 3

## Data Availability

The data presented in this study are available on request from the corresponding author. The data are not publicly available due to privacy.
